# Integration of Metabolome and Transcriptome Studies Reveals Flavonoids, Abscisic Acid, and Nitric Oxide Comodulating the Freezing Tolerance in *Liriope spicata*

**DOI:** 10.3389/fpls.2021.764625

**Published:** 2022-01-27

**Authors:** Zhen Peng, Ye Wang, Wen-Tian Zuo, Yue-Rong Gao, Run-Zhi Li, Chun-Xin Yu, Zi-Yan Liu, Yi Zheng, Yuan-Yue Shen, Liu-Sheng Duan

**Affiliations:** ^1^College of Plant Science and Technology, Beijing University of Agriculture, Beijing, China; ^2^Beijing Key Laboratory for Agricultural Application and New Technique, Beijing, China; ^3^Bioinformatics Center, Beijing University of Agriculture, Beijing, China

**Keywords:** *Liriope spicata*, freezing stress, metabolomics, transcriptomics, flavonoids, ABA, NO

## Abstract

*Liriope spicata* is an evergreen perennial ornamental groundcover with a strong freezing tolerance. However, the molecular mechanism underlying the freezing tolerance in *L. spicata* remains unclear. In this study, a comprehensive investigation of *L. spicata* freezing tolerance was conducted at the levels of physiology and biochemistry, metabolite, and transcript during the stress treatment. There were 581 unique differentially expressed metabolites (DEMs) and 10,444 unique differentially expressed genes (DEGs) between freezing treatment and normal cultured plant in leaves. Integrated analysis of metabolomics and transcriptomics showed that flavonoid biosynthesis, carbohydrate metabolism, amino acid metabolism, lipid metabolism, and signal transduction pathways were prominently enriched in response to the freezing stress in *L*. *spicata*. Now, we identified genes and metabolites involved in the flavonoid pathway, abscisic acid (ABA) biosynthesis, and the oxidative synthesis pathway of nitric oxide (NO), which may form a regulatory network and play a synergistic effect in osmotic adjustment, reactive oxygen species (ROS) homeostasis, and stomatal closure under freezing stress. These results offer a comprehensive network of flavonoids, ABA, and NO comodulating the freezing tolerance in *L. spicata*.

## Introduction

Freezing stress (<0°C) is a primary factor that determines plant geographic distribution and restrains the choice of plant species in the landscape design of the ground cover. The freezing temperature forces the leaves of most groundcovers to wither, reducing the ornamental value and resulting in a large area of exposed soil, causing ecosystem problems such as flying dust. To solve the contradiction between the green landscape and the cold winter environment in the north, one of the key technologies urgently needed is to study and make full use of the rural groundcover resources with freezing tolerance. *Liriope spicata* is an evergreen perennial, tufted, or rhizomatous ornamental groundcover in the *Asparagaceae* family. *L. spicata* is widely distributed and cultivated in vast areas of China, Japan, and Vietnam and introduced in several other countries, including the United Kingdom and United States. *L. spicata* has a strong freezing tolerance (able to survive the winter safely with green leaves even at −15°C outdoor temperature) and is the main groundcover in the cold winter in the northern China that forms green landscapes and suppresses ground dust. However, the mechanism underlying the freezing tolerance in *L*. *spicata* remains largely unknown.

Survival under cold stress requires the integration of adaptive metabolic, physiological, and molecular responses that are tightly associated with stress-related gene expression, enzyme activities, and the concentrations of primary and secondary metabolites (Chinnusamy et al., 2007; Guo et al., 2018; Ding et al., 2020). Cold stress affects membrane fluidity, thereby altering the structure and activity of membrane-localized proteins such as calcium (Ca^2+^) channels (Yuan et al., 2018). Then, the cold signal is transduced to a regulatory network and triggers multiple corresponding cold responses in the cell (Guo et al., 2018; Yuan et al., 2018). The C-repeat binding factor/dehydration-responsive element-binding protein 1 (CBF/DREB1) dependent transcriptional regulatory pathway is essential for plant responses to cold stress (Jia et al., 2016; Shi et al., 2018). *CBF*/*DREB1* is rapidly upregulated by cold stress and then activates the transcription of cold-responsive (*COR*) genes by binding to their promoters (Stockinger et al., 1997; Jia et al., 2016). Mitogen-activated protein kinase (MAPK) cascades, transcription factors (TFs), reactive oxygen species (ROS), flavonoids, nitric oxide (NO), and phytohormones such as abscisic acid (ABA) also play pivotal roles in the cold response process in plants (Shinkawa et al., 2013; Simontacchi et al., 2015; Schulz et al., 2016; Li H. et al., 2017; Zhao et al., 2017; Chen et al., 2018; Zhang et al., 2020).

Abscisic acid signaling plays important roles in plant development and adaptation to environmental stress. In the seedling stage, the transgenics of ABA receptor *PYL10*-overexpressed rice plants show a significantly higher survival rate under cold stress as compared with WT plants (Verma et al., 2019). MYB96-HHP (heptahelical protein) module integrates ABA-dependent and ABA-independent signals and activates the CBF pathway, ensuring plant adaptation to cold stress (Lee and Seo, 2015). Open stomata 1 (OST1)/sucrose non-fermenting 1 (SNF1) related protein kinase 2.6 (SnRK2.6) is a Ser/Thr protein kinase, which is activated by ABA and cold. *OST1*-overexpressing plants were more resistant, and *ost1* knockout lines were hypersensitive to freezing (Mustilli et al., 2002). ABA also interrelates with NO signaling. In guard cells, NO negatively regulates ABA signaling by inhibiting OST1/SnRK2.6 through S-nitrosylation (Wang et al., 2015).

Flavonoids are the main secondary metabolites that are involved in the plant defense abiotic response. In *Arabidopsis thaliana*, flavonoids are regarded as the determinants of freezing tolerance and cold-acclimation (CA) using the 20 mutant lines that are affected in the different steps of the flavonoid biosynthetic pathway (Schulz et al., 2016). The winter ecotype of *Brassica napus* is more resistant to low temperature compared to the spring ecotype. In the metabonomic analysis of the *B. napus*, it was found that the content of flavonol metabolites such as quercetin and dihydromyricetin in the seedlings significantly increased after cold stress (Jian et al., 2020). In apple (*Malus domestica*), the accumulation of flavonols promotes the scavenging of ROS and plant survival under drought conditions (Wang et al., 2020). In tomato (*Solanum lycopersicum*), flavonols reduce the accumulation of ROS and modulate the ABA-dependent ROS burst in guard cells, facilitating stomatal opening to modulate leaf gas exchange (Watkins et al., 2017).

Nitric oxide is a small, gaseous signaling molecule that has been studied for decades in mammalian cells. Recent research findings have shown that NO regulates plant growth, enhances nutrient uptake, and activates stress tolerance mechanisms in most plants (Sun et al., 2021). NO is a negative regulator of the chlorophyll (Chl) catabolic pathway and positively maintains the stability of thylakoid membranes during leaf senescence (Liu and Guo, 2013). In tomato, an exogenous NO donor, sodium nitroprusside (SNP), protects the photosynthetic system from damage, thereby enhancing chilling tolerance (Diao et al., 2016). In tea plants (*Camellia sinensis* L.), exogenous NO increased flavonoid concentrations by promoting the transcription and activity of the flavonoid biosynthesis enzyme phenylalanine ammonia-lyase (PAL) in leaves (Li X. et al., 2017). NO is considered as a critical component of mediating phytohormonal actions, interacting with ROS, and modulating gene expression and protein activity under abiotic stress conditions. NO deficiency decreases the plant CA response, alters ABA content and signaling, and increases anthocyanin accumulation (Costa-Broseta et al., 2019). Emerging evidence implies that NO regulates plant growth, enhances nutrient uptake, and activates disease and stress tolerance mechanisms in most plants, making NO a potential tool for use in improving the yield and quality of horticultural crop species (Simontacchi et al., 2015; Sun et al., 2021).

In this study, we aimed to explore the molecular mechanisms at the transcriptome and metabolome levels from a wide perspective and sought the clues linking genes and marker metabolites in response to freezing stress. We globally analyzed the metabolic pathways involved in differentially expressed genes (DEGs) and metabolites when suffering freezing. In addition, we proposed a network formed by flavonoid biosynthesis, the ABA signaling pathway, and NO signaling, which comodulated the freezing tolerance in *L. spicata*.

## Materials and Methods

### Plant Materials

Experiments were conducted in the greenhouse of Beijing University of Agriculture. The *L. spicata* accession BUA1032 was collected from the germplasm nursery after 2 years of good planting. The plants were divided into clusters of three buds and transplanted into containers using a 8-L loam-peat-roseite substrate. The plants were grown at 25/20°C (day/night) temperature and 12/12 h (light/dark) photoperiods with fluorescent white light at 200 μmol m^–2^ s^–1^.

### Freezing Stress Assays

The freezing tolerance program was performed in a freezing chamber. Before freezing stress, the plants were pretreated at 4°C for 5 days [cold-acclimated (CA)] or without cold treatment [non-acclimated (NA)], and the soil humidity was controlled at 40∼50%. The freezing program began at 0°C, and then the temperature was dropped by 1°C per h until reaching −15°C. The temperature remained stable for 24 h, and samples were taken at 0, 2, 6, 12, and 24 h of −15°C freezing stress. After freezing treatment, the plants were then shifted to 4°C for 12 h before being transferred to 25°C normal conditions for 3 days of recovered growth. All materials were analyzed in three biological replicates in this study.

### RNA Isolation and Transcriptomics Analysis

*Liriope spicata* samples were collected from control (25°C) and freezing-treated leaves (at 0, 2, 6, 12, and 24 h of −15°C freezing stress with CA) and quickly immersed in liquid nitrogen. Then, the high-quality total RNA (three biological replicates for each treatment) was isolated from liquid nitrogen frozen leaves using the TRIzol method, and high-throughput sequencing was implemented on an Illumina HiSeq 2500 platform. After filtering raw data, the transcripts were assembled with the Trinity software (Grabherr et al., 2011) to obtain the long-contig transcriptome. Seven databases [NR, NT, Pfam, SwissProt, GO, Kyoto Encyclopedia of Genes and Genomes (KEGG), and KOG] were used for annotating the gene function. The longest transcript in each gene was regarded as a unigene. Plant TFs were predicted using the iTAK software^1^. The levels of gene expression were estimated by the fragments per kilobase of exon per million mapped reads (FPKM). DEGs were determined using DESeq2, with a screening threshold of |log2(Fold Change)| > 1 and *p*-adj < 0.05 (Love et al., 2014). These DEGs were then used for enrichment analyses of the KEGG pathway^2^.

### Metabolite Extraction and Quasi-Targeted Metabolomics Analysis

Leaf material (six biological replicates for each treatment) was extracted two times with extraction solvent methanol/water [8:2 (v/v)] containing 0.1% formic acid at 0°C for 30 min. The solution was centrifuged at 12,000 rpm for 10 min at 4°C, and the supernatant samples were filtered with a 0.22-μm filter sterilizer before liquid chromatography coupled to electrospray tandem mass spectrometry (LC-MS/MS) analysis. Each experimental samples were taken in equal volumes and blended as QC samples. Blank sample is 53% methanol aqueous solution containing 0.1% formic acid instead of an experimental sample, the pretreatment process is the same as an experimental sample.

Liquid chromatography coupled to electrospray tandem mass spectrometry analyses were performed using an ExionLC™ AD system (SCIEX) coupled with a QTRAP ^®^ 6500+ mass spectrometer (SCIEX) in Novogene Co., Ltd. (Beijing, China). For the positive polarity mode, samples were injected onto a BEH C8 Column (100 mm × 2.1 mm, 1.9 μm) using a 30 min linear gradient at a flow rate of 0.35 ml min^–1^. The eluents were eluent A (0.1% formic acid-water) and eluent B (0.1% formic acid-acetonitrile). The solvent gradient was set as follows: 5% B, 1 min; 5–100% B, 24.0 min; 100% B, 28.0 min; 100–5% B, 28.1 min; and 5% B, 30 min. For the negative polarity mode, samples were injected onto a HSS T3 Column (100 mm × 2.1 mm) using a 25 min linear gradient at a flow rate of 0.35 ml min^–1^. The eluents were eluent A (0.1% formic acid-water) and eluent B (0.1% formic acid-acetonitrile). The solvent gradient was set as follows: 2% B, 1 min; 2–100% B, 18.0 min; 100% B, 22.0 min; 100–5% B, 22.1 min; and 5% B, 25 min. QTRAP ^®^ 6500+ mass spectrometer was operated in a positive polarity mode with curtain gas of 35 psi, collision gas of medium, ion spray voltage of 5,500 V (positive) or −4,500 V (negative), temperature of 500°C, ion source gas of 1: 55, and ion source gas of 2: 55.

Based on the Novogene database, the sample metabolite detection was implemented in the multiple reaction monitoring (MRM) mode of the SCIEX QTRAP ^®^ 6500+ mass spectrometer. The compounds were quantified according to Q3 (production), and qualitative analysis was performed according to the retention time of the detected substance, the information of the production pair (Q1/Q3), and the secondary spectrum data. The data were analyzed by a principal component analysis (PCA), partial least squares-discriminant analysis (PLS-DA), and other multivariate statistical analyses to elucidate differences in metabolites between the samples.

### Integrated Analysis of Metabolome and Transcriptome

The correlation analysis between the significantly different genes obtained by transcriptome analysis and the significantly different metabolites obtained by metabolomics analysis was based on the Pearson correlation coefficient to measure the degree of correlation between the different genes and the different metabolites. To determine the main biochemical pathways and signal transduction pathways in which differential metabolites and differential genes participate together, all differential genes and metabolites obtained were simultaneously mapped to the KEGG pathway database to obtain their common pathway information.

### Quantitative PCR Assays

The RNA samples used for quantitative PCR (qPCR) assays were exactly the same as those used for RNA-sequencing (RNA-seq) assays. Then, the high-quality total RNA was reverse transcribed using a FastKing One Step RT-PCR kit (Tiangen, China; Cat#KR123). qPCR was performed with TB Green ^®^ Premix Ex Taq™ II (Takara, Japan; Cat#RR820A) on a CFX96 Touch Real-Time PCR Detection System (Bio-Rad, Hercules, CA, United States). The *ACT1* (Cluster-21629.42877) gene of *L. spicata* was used as an internal reference gene. The gene relative expression levels were calculated by the 2^(−ΔΔCT^) method. All experiments were performed in three biological replicates. The primers used for qPCR analysis in this study are listed in Supplementary Table 1.

### Analysis of Water Status in Leaf

The content of free water (FWC) and bound water (BWC) in plants is closely related to the existence of the cytoplasm. The refractometer method was used to detect the FWC and BWC of the samples. The leaves of *L. spicata* were collected at 9–10 a.m. and divided into two parts. One part was measured for the fresh weight (*W*_f_) and dry weight (*W*_d_) after being baked at 105°C to constant weight. The leaf total water content (TWC) was calculated as (*W*_f_−*W*_d_)/*W*_f_ × 100%. The other part (*W*_f1_) was completely immersed in 10 ml sucrose solution (concentration of C_1_, weight of *W*). After sufficient water exchange, the concentration was measured as C_2_. FWC was calculated as *W* × (*C*_1_ – *C*_2_)/(*W*_f1_ × *C*_2_) × 100%, and BWC was calculated as TWC – FWC.

### Ion Leakage Assays

Ion leakage is a symptom of cold-induced membrane damage and is used as an indicator of freezing tolerance. Ion leakage assays were carried out as described in Lee and Zhu (2010) with slight modifications. The banded leaflets were quickly crosscut with a surgical blade and placed in 15-ml test tubes containing 10 ml of CO_2_-free deionized water. The initial electrical conductivity value was recorded as *S*_0_. The samples were shaken at 22°C for 1 h, and the conductivity was measured as *S*_1_. After a water bath at 100°C for 30 min, the samples were shaken and cooled to 22°C, and the value was detected as *S*_2_. Ion leakage was calculated as (*S*_1_ – *S*_0_)/(*S*_2_ – *S*_0_) × 100%.

### Measurement of Chl Relative Content

The leaf Chl relative content was measured by Chl meter SPAD-520 Plus (Konica Minolta, Marunouchi, Japan). The freezing treated plants were recovered at 25°C for 3 days before measuring Chl relative content. Each set of processed samples collected 50 data.

### Determination of Antioxidant Enzyme Activity

After the freezing process, fresh leaves were immediately sampled to detect the antioxidant enzyme activity, including superoxide dismutase (SOD), peroxidase (POD), and catalase (CAT). The measurements were conducted using the SOD assay kit (Solarbio, China; Cat#BC0170), POD assay kit (Solarbio, China; Cat#BC0090), and CAT assay kit (Solarbio, China; Cat#BC0200) following the manufacturer’s protocols. A UV-6000PC UV-Visible Spectrophotometer (Shanghai, China) was used to measure the absorbance values. Six biological replicates for each sample were carried out through the whole process.

### Stomatal Aperture Assays

Microscopy images of stomatal phenotypes were obtained from freshly fixed samples and visualized using an electron fluorescence microscope (Olympus BX53, Tokyo, Japan). The abaxial epidermises of the living leaf were torn off with tweezers and quickly fixed in absolute ethanol for 3 min. The stomata fluorescence was detected with an excitation wavelength of 488 nm. The stomatal aperture (width) was determined from the measurements of 40–60 stomata per treatment. Each experiment was replicated three times.

## Results

### Physiological and Biochemical Responses to Freezing Stress

**FIGURE 1:**
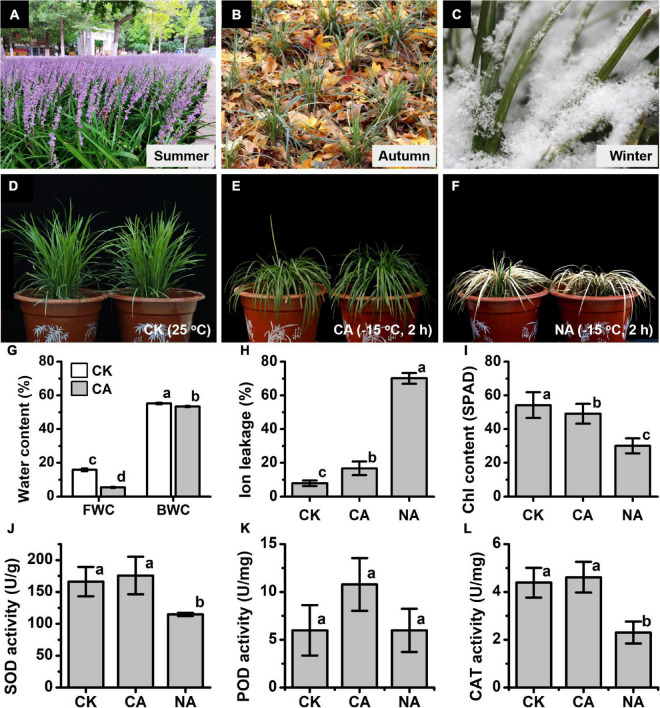
Physiological and biochemical responses to the freezing stress in *Liriope spicata*. **(A)** Flowering landscape in summer. **(B)** Leaves of *L. spicata* do not turn yellow in autumn. **(C)** Leaves remain fresh and green when covered by heavy snow in winter. **(D)** Normal phenotype under 25°C (CK) condition. **(E)** Freezing phenotype under cold-acclimation (CA) conditions. **(F)** Freezing phenotype under non-acclimation (NA) conditions. **(G)** Water physiology. FWC, free water content; BWC, bound water content. **(H)** Ion leakage. **(I)** chlorophyll (Chl) relative content. **(J)** superoxide dismutase (SOD) enzyme activity. **(K)** Peroxidase (POD) enzyme activity. **(L)** Catalase (CAT) enzyme activity. CK, control samples that culture under 25°C; CA, freezing treatment samples under –15°C 2 h with 4°C CA for 3 days priority; NA, freezing treatment samples under –15°C 2 h without 4°C CA. The letters a,b,c in **(G–L)** represent significant differences among the samples; Student’s *t*-test, *p* < 0.05.

To evaluate the freezing tolerance in *L. spicata* accession BUA1032, we identified the phenotype of the materials cultivated in the non-protected field and greenhouse. The leaves are dark green in autumn and maintain viability, and can survive the winter safely (the lowest temperature reached −15°C or even below). *L. spicata* can maintain good plant morphology even when covered by heavy snow in winter (Figures 1A–C). In a controlled environment, −15°C freezing treatment was carried out under either CA or NA conditions. After 3 days of recovery of growth, the leaves of the freezing plants with CA endured the stress and remained green, but the quick-freezing leaves exhibited severe damage (Figures 1D–F).

The water status of the leaves was tested, and the results showed that the control leaves grown at 25°C contained 15.85% FWC and 55.22% BWC, while the leaves upon freezing treatment with CA contained 5.41% FWC and 53.40% BWC (Figure 1G). This result suggested that the prominent forms of high BWC and high ratio of BWC/FWC might have important roles in the freezing tolerance ability of *L. spicata*. The leaves upon freezing treatment with CA showed increased ion leakage compared to those upon treatment with CK (Figure 1H). Although the change reached a significant level between the leaves of CK (7.92%) and CA (16.76%), it was still safe for this plant. However, the ion leakage of quick-freezing samples (NA, −15°C, 2 h) increased to 70.13%, which reached a lethal degree (Figure 1H). After recovering at 25°C for 3 days, the CA plants still hold about 50 SPAD Chl content to keep the leaves green (Figure 1I). The determination of antioxidant enzyme activity showed that SOD, POD, and CAT were responded to freezing stress, which displayed higher levels in the CK and CA leaves compared to the NA (Figures 1J–L). These results indicate that *L. spicata* under CA conditions has a strong freezing tolerance to extreme low temperatures.

### Transcriptomics Analysis of *L. spicata* Leaves in Response to Freezing Stress

**FIGURE 2:**
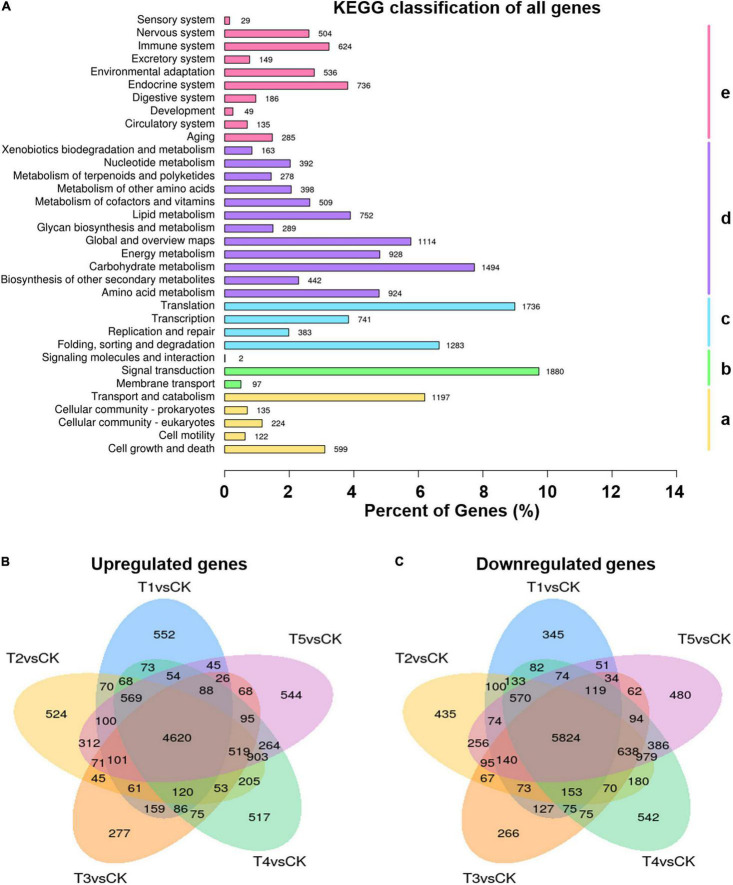
Global transcriptome of plants in response to freezing stress. **(A)** KEGG classification of all genes annotated. (a) Cellular processes, (b) Environmental information processing, (c) Genetic information processing, (d) Metabolism, (e) Organismal systems. **(B,C)** Venn diagrams showing the overlap among upregulated **(B)** and downregulated **(C)** differentially expressed genes (DEGs) in each of the different duration time freezing treatments and control. T1, –15°C 0 h under CA condition; T2, –15°C 2 h under CA condition; T3, –15°C 6 h under CA condition; T4, –15°C 12 h under CA condition; and T5, –15°C 24 h under CA condition. Numbers above each bar and each oval represent gene numbers.

To understand the molecular basis of the freezing tolerance in *L. spicata*, transcriptomics analysis was used to identify the DEGs in the leaves treated with −15°C freezing (CA before treatment) for duration times of 0, 2, 6, 12, and 24 h and control samples treated at 25°C. In this study, a total of 135.9 million clean reads were produced, and 315,382 transcripts were assembled through Trinity; 110,639 unigenes were functionally annotated. These genes were enriched mainly in cellular processes, environmental information processing, genetic information processing, metabolism, and organismal systems (Figure 2A). With the DESeq2 software and the filter criteria | Log2FC| ≥ 1, *p*-adj < 0.05, the DEGs were identified by a pairwise comparison between freezing treatment and control samples of *L. spicata* leaves (Supplementary Table 1). Furthermore, the vast majority of DEGs shared 4,620 upregulation and 5,824 downregulation between each freezing treatment and the control (Figures 2B,C), suggesting that a potentially common molecular mechanism is involved in the response to freezing stress.

Comparing DEGs between freezing samples and the control, KEGG enrichment analyses showed that the upregulated DEGs were specifically enriched in the pathways of “flavonoid biosynthesis (ko00941),” “flavone and flavonol biosynthesis (ko00944),” “fructose and mannose metabolism (ko00051),” “ascorbate and aldarate metabolism (ko00053),” “arginine and proline metabolism (ko00330),” and “cutin, suberin, and wax biosynthesis (ko00073)” (Supplementary Table 2). These results suggested that *L. spicata* accumulates osmotic adjustment substances to maintain cell homeostasis and produces a thickened cell surface protection layer to achieve enhanced freezing resistance. The downregulated DEGs were abundant in “photosynthesis–antenna proteins (ko00196),” “plant–pathogen interaction (ko04626),” “linoleic acid metabolism (ko00591),” “alpha-linolenic acid metabolism (ko00592),” “fatty acid elongation (ko00062),” and “tryptophan metabolism (ko00380)” (Supplementary Table 3). These results imply that freezing stress restricts photosynthesis and prevents the degradation of unsaturated fatty acids. At the same time, the metabolism of tryptophan, the precursor of auxin biosynthesis, is also inhibited by the induction of freezing.

As TFs can regulate the expression of other genes and play an important role in regulating plant growth and increasing the freezing tolerance in plants, we further analyzed the expression profiles of TFs in all the samples. A total of 2,047 TFs were identified in the leaves exposed to low temperatures. Among these TFs, there were 830 genes whose expression level had changed significantly in at least one pairwise relationship that compared freezing to control samples, with 312 upregulated and 518 downregulated. These TFs were categorized into 20 main TF families, and most of these were abiotic stress-related families, such as AP2/ERF, bHLH, bZIP, HSF, MADS, MYB, NAC, and WRKY (Supplementary Table 4). There were 135 TFs upregulated in all the five freezing phases compared with the TFs at normal temperature, while 262 downregulated TFs were identified (Supplementary Table 4). These results provide some useful suggestions for further study of the functions of TFs in *L. spicata* freezing resistance.

### Metabolomics Analysis Identifies Flavonoids as the Main Secondary Metabolites That Changed in *L. spicata* Leaves Under Freezing Stress

**FIGURE 3:**
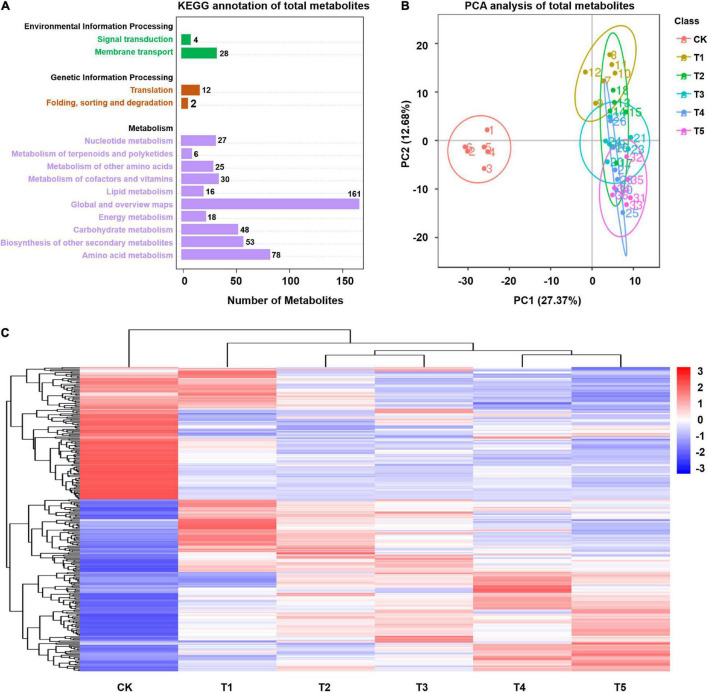
Global metabolome of plants in response to freezing stress. **(A)** KEGG classification of total metabolites annotated. Numbers above each bar represent metabolite numbers. **(B)** Principal component analysis (PCA) displays the divergence of the respective metabolomes in response to freezing stress and the control. **(C)** Differentially expressed metabolites (DEMs) were hierarchically clustered (Euclidean distance) based on their abundance under freezing stress relative to control samples.

Leaf tissues of *L. spicata* with normal culture and freezing treatment were subjected to metabolomics assays using high-performance liquid chromatography with tandem mass spectrometric (HPLC-MS/MS). This analysis identified 581 metabolites in leaves. *Via* the KEGG pathway annotation, the metabolites were classified mainly as environmental information processing (32 annotated), genetic information processing (14 annotated), and metabolism (462 annotated) (Figure 3A). The PCA plot showed that the five freezing treatments were clearly separated from the control by PC1 and represented the altered accumulation of chemically related metabolites when suffering freezing stress (Figure 3B). By the criteria of a PLS-DA model VIP > 1.0, FC > 2.0 or FC < 0.5, and *p* < 0.05, 156, 169, 167, 165, and 190 significantly differentially expressed metabolites (DEMs) were detected in *L. spicata* leaves with five different lengths of freezing duration treatment (−15°C) compared with the control (25°C). Hierarchical clustering analysis showed that the enrichment patterns of freezing sample libraries were classified into the same cluster (Figure 3C). The KEGG pathway analysis of DEMs showed that these detections highlighted the enrichment in carbon fixation in photosynthetic organisms (map00710), flavone and flavonol biosynthesis (map00944), flavonoid biosynthesis (map00941), phenylpropanoid biosynthesis (map00940), and lysine biosynthesis (map00300) (*p* < 0.05; Supplementary Table 5).

### Accumulation of Flavonols in *L. spicata* Leaves Promotes Resistance to Freezing Stress

We screened the total DEMs and investigated the top 20-fold change metabolites. As a result, we found that 10 individual flavonoids were significantly upregulated in the freezing samples (FC > 5 and *p* < 1 × 10^–3^). Rutin and two kaempferol glycosides were upregulated more than 10-fold in all the five freezing samples compared with the control.

**FIGURE 4:**
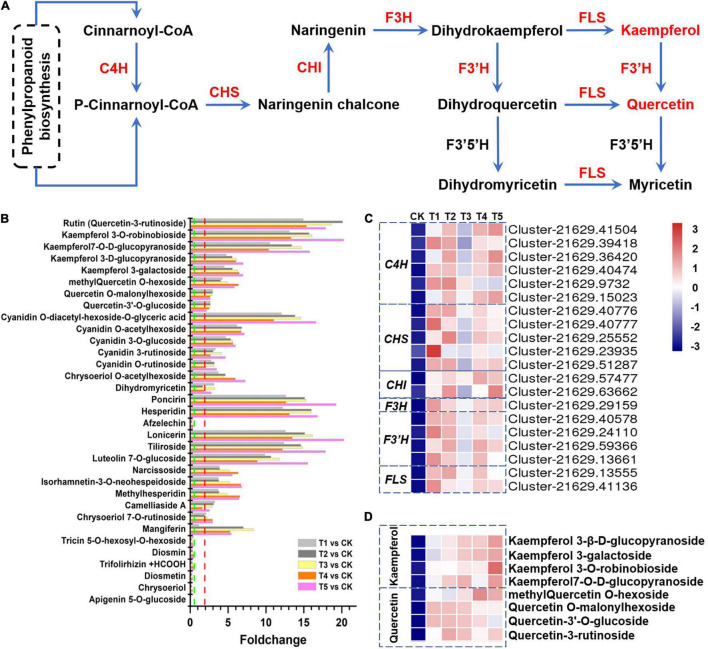
Flavonoids in response to freezing stress. **(A)** Flavonol biosynthesis pathway and genes involved in this process. **(B)** The fold changes of flavonoid accumulation in plants subjected to each freezing stress compared with the control, including 26 upregulated and 7 downregulated flavonoid compounds. **(C)** The flavonol biosynthesis-associated genes were hierarchically clustered (Euclidean distance) based on their expression level. **(D)** The flavonols were hierarchically clustered (Euclidean distance) based on their abundance. C4H, cinnamate 4-hydroxylase; CHS, chalcone synthase; CHI, chalcone isomerase; F3H, flavanone 3-hydroxylase; F3’H, flavonoid 3’-hydroxylase; FLS, flavonol synthase.

In this study, a total of 83 different flavonoids, including 26 upregulated metabolites and 7 downregulated metabolites (VIP > 1.0, FC > 2.0 or FC < 0.5, and *p* < 0.05), were identified to have significantly different accumulation after freezing stress. The fold changes of 33 flavonoids with significant differences in enrichment are shown in Figure 4A. Among these flavonoids, rutin and kaempferol 3-O-robinobioside showed the highest fold changes, with 14.9- to 20.1-fold and 13.1- to 20.1-fold, respectively (Figure 4B). Most genes and enzymes involved in the pathway lead to this chemical shift. Surprisingly, the flavonol biosynthesis pathway genes *C4H* (six transcripts), *CHS* (five transcripts), *CHI* (two transcripts), *F3H* (one transcript), *F3’H* (four transcripts), and *FLS* (two transcripts) consistently showed significantly increased expression levels when the plants were exposed to freezing stress (Figures 4A,C). We detected four types of quercetins (rutin, methylquercetin O-hexoside, quercetin O-malonylhexoside, and quercetin-3′-O-glucoside) and four types of kaempferols (kaempferol 3-O-robinobioside, kaempferol 7-O-D-glucopyranoside, kaempferol 3-D-glucopyranoside, and kaempferol 3-galactoside) were significantly enriched in flavonols under freezing treatments (Figure 4D). These results indicate that the flavonol biosynthesis pathway positively responds to the freezing stress in *L. spicata*.

### Integrated Analysis of Abscisic Acid Responding to the Freezing Stress in *L. spicata*

**FIGURE 5:**
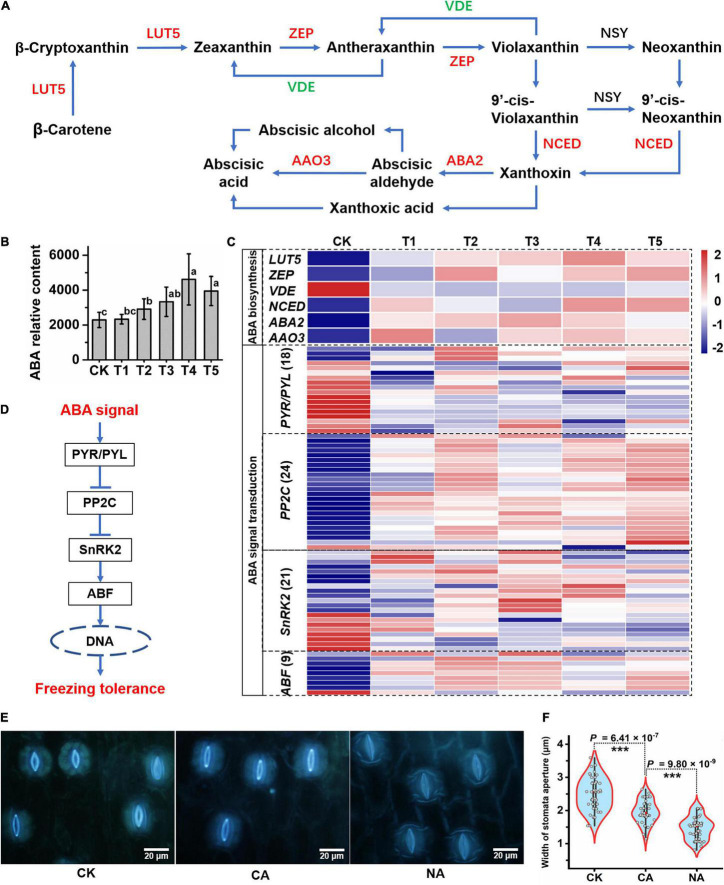
The response of abscisic acid (ABA) biosynthesis to freezing stress. **(A)** The biosynthesis pathway of ABA was produced according to the KEGG database. LUT5, beta-ring hydroxylase; ZEP, zeaxanthin epoxidase; VDE, violaxanthin de-epoxidase; NCED, 9-cis-epoxycarotenoid dioxygenase; ABA2, xanthoxin dehydrogenase; AAO3, abscisic-aldehyde oxidase. **(B)** ABA relative content detected by liquid chromatography coupled to electrospray tandem mass spectrometry (LC-MS/MS) in each sample. The letters represent significant differences among the samples; Student’s *t*-test, *p* < 0.05. **(C)** The identified ABA biosynthesis-associated genes and ABA signal transduction genes were hierarchically clustered (Euclidean distance) based on their expression level. **(D)** The pathway of ABA signaling transduction in plants. PYR1/PYL, pyrabactin resistance 1/PYR1-like; PP2C, type 2C protein-phosphatase; SnRK2, sucrose non-fermenting 1-related protein kinase subfamily 2; ABF, ABA-responsive element binding factors. **(E)** Stomatal closure and apertures of leaf abaxial epidermis are shown under control and freezing stress conditions. **(F)** Mean ± SD of 40–60 stomata from the four separate experiments are reported as the width of stomatal aperture. Asterisks represent significant differences in stomatal aperture (*p* < 0.001; Student’s *t*-test) between freezing-treated leaves and the control.

Given that ABA is important in the response to abiotic stress in plants, we integratively analyzed changes in ABA biosynthesis and signaling pathway gene expression and ABA content. We found that most genes related to the ABA biosynthetic pathway, such as *LUT5* (beta-ring hydroxylase), *ZEP* (zeaxanthin epoxidase), *NCED* (9-cis-epoxycarotenoid dioxygenase), *ABA2* (xanthoxin dehydrogenase), and *AAO3* (abscisic-aldehyde oxidase), showed a significantly increased expression under freezing stress (Figure 5A). Moreover, the expression of the violaxanthin de-epoxidase (*VDE*) gene, which catalyzes the conversion of violaxanthin to zeaxanthin at the lumen side of the thylakoids, was decreased (Figures 5A,C). The expression patterns of these genes suggest that the pathway is conducive to the enrichment of ABA. The LC-MS results showed that the ABA content continued to increase and reached an extremely significant level of enrichment at T2 and later periods of freezing treatment, with a 1.5–2-fold increase (Figure 5B). Genes involved in the ABA signal transduction pathway responded to the freezing stress in *L. spicata*. In this study, 18 ABA receptors, pyrabactin resistance 1/PYR1-like (PYR/PYL), were differentially expressed, with most of them downregulated when suffering freezing stress. Twenty-four PP2Cs (type 2C protein-phosphatases) were detected, and most were upregulated by freezing. Twenty-one SNF1-related protein kinase subfamily 2 (SnRK2) genes were identified, and most were significantly induced. Eight of nine ABA-responsive element binding factor (ABF) genes displayed increased expression under freezing stress (Figures 5C,D).

One of the regulating factors of stomatal closure is the increase in ABA content in the plant. Here, we examined stomatal closure and measured the stomatal aperture (Figures 5E,F). The abaxial epidermis of leaves was quickly torn off and immediately put into absolute ethanol to fix the cell morphology after treatment. Under a fluorescence microscope, the CK (25°C) epidermis displayed opening stomata, and CA (−15°C 2 h) showed significantly narrower opening stomata (Figures 5E,F). However, the stomata in the quickly freezing samples of NA (−15°C 2 h) were almost closed (Figures 5E,F). These results illustrate an inverse relationship between the level of ABA and stomatal aperture, which are linked to altered rates of an ABA-dependent stomatal closure under freezing stress.

### The Nitric Oxide Signaling Pathway May Regulate Compound Metabolism Under Freezing Stress

**FIGURE 6:**
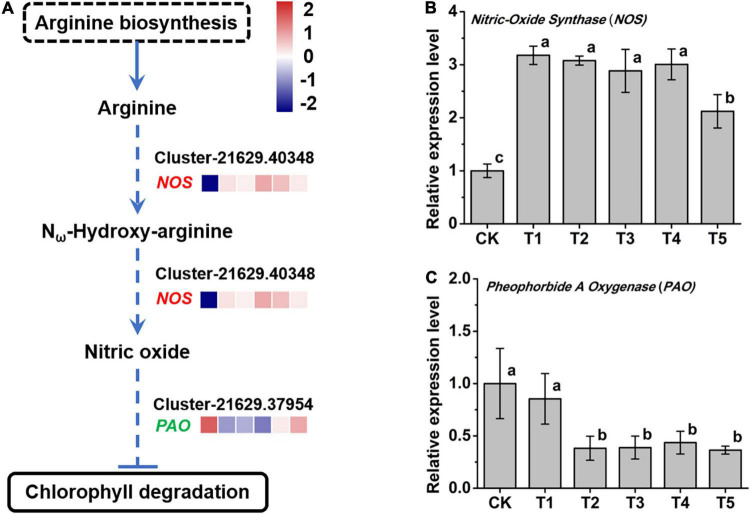
The response of nitric oxide (NO) metabolism to freezing stress. **(A)** The biosynthesis pathway of NO was produced according to the KEGG database. **(B)** The expression of *NOS*, a NO synthase gene, was upregulated under freezing stress. **(C)** The expression of *Pheophorbide a Oxygenase* (*PAO*), a key gene in the Chl degradation pathway, was downregulated under freezing stress. The letters in **(B)** and **(C)** represent significant differences among the samples; Student’s *t*-test, *p* < 0.05.

In the integrated analysis, we found that the arginine biosynthesis pathway (ko00220) and arginine metabolism pathway (ko0330) were modulated by freezing stress. What attracted attention was that the oxidative pathway of NO synthesis by oxidizing arginine was upregulated (Figure 6A). The nitric oxide synthase (*NOS*, Cluster-21629.40348) gene, which encodes an enzyme that catalyzes arginine to NO, was significantly increased after the freezing stress in leaves (Figure 6B). A previous study found that NO inhibits the degradation of Chl (Liu and Guo, 2013). In this study, the expression of the *Pheophorbide a Oxygenase* (*PAO*, Cluster-21629.37954) gene, a key control point in the overall regulation of Chl degradation, was significantly reduced after 4 h freezing stress (Figure 6C) and negatively correlated (Pearson correlation coefficient *r* = − 0.52) with the expression of the *NOS* gene. These results indicate that the degradation process of Chl was inhibited, which may explain the characteristics of *L. spicata* to stay green over the winter.

## Discussion

### Molecular Networks Involved in the Freezing Tolerance in *L. spicata*

In this study, we performed an integrated analysis of the transcriptome and metabolome profiled from *L. spicata* leaves cultured at normal temperature and suffered from the freezing stress of five different durations. We identified 10,444 DEGs and 581 DEMs between the five freezing samples and the control, suggesting that freezing stress is a critical factor for transcriptomics and metabolomics data. The DEGs and DEMs were coenriched mainly in the pathways of carbohydrate metabolism, amino acid metabolism, cutin, suberin and wax biosynthesis, phytohormone biosynthesis, and secondary metabolite biosynthesis, such as flavonoid biosynthesis and stilbenoid, diarylheptanoid, and gingerol biosynthesis. These results suggested that freezing stress caused the comprehensive defense response of *L. spicata*, including the enrichment of osmotic adjustment substances and antioxidant compounds and signal transduction of phytohormones. ABA and indole-3-acetic acid (IAA) are important signaling molecules that regulate metabolic processes related to stress adaptation and induce stress resistance in plants (Verma et al., 2019). Numerous studies have shown that the ABA content increases under abiotic stress and induces the expression of downstream genes (Vaidya et al., 2019). The upregulated cellular defensive substance synthesis pathways supported the maintenance of homeostasis in the cellular membrane to achieve enhanced freezing resistance (Liang Y. et al., 2020). Meanwhile, the downregulated DEGs were abundant in “photosynthesis–antenna proteins,” “plant–pathogen interaction,” “linoleic acid metabolism,” “alpha-linolenic acid metabolism,” “fatty acid elongation,” and “tryptophan metabolism,” implying that freezing stress reduced photosynthetic activity and IAA biosynthesis by the tryptophan pathway, which may constitute an energy-saving strategy under low CO_2_ availability (Guo et al., 2021).

Transcription factors regulate the expression of other genes and play important roles in regulating plant growth and increasing cold tolerance in plants (Shi et al., 2018). In this study, we identified 830 TF genes that displayed a significantly different expression. These genes were classified mainly into the AP2/ERF, bHLH, bZIP, HSF, MADS, MYB, NAC, and WRKY families. In bermudagrass [*Cynodon dactylon* (L.) Pers], *CdERF1* is induced by cold, drought, and salinity stresses. The overexpression of *CdERF1* activates a subset of stress-related genes in transgenic *Arabidopsis*, such as *CBF2*, *pEARLI1* (lipid transfer protein), *PER71* (POD), and lipid transfer protein (*LTP*), suggesting that *CdERF1* may be an ideal candidate in the effort to improve cold tolerance (Hu et al., 2020). In sweet orange (*Citrus sinensis*), most of the *CsbHLH* genes are responsive to cold stress, and the greatest upregulation gene *CsbHLH18* functions in the modulation of cold tolerance and the homeostasis of ROS by regulating antioxidant genes (Geng and Liu, 2018). *AtMYB15* is a transcriptional repressor of cold signaling because it negatively regulates *AtCBF3*, which is required for the freezing tolerance in *Arabidopsis*. Meanwhile, AtMPK6-induced phosphorylation reduces the affinity of AtMYB15 binding to the At*CBF3* promoter (Kim et al., 2017). The homeostasis of the negative regulator MYB15 is controlled by an OST1-PUB25/26 module regulating the duration and amplitude of the cold response (Wang et al., 2019). Thus, TFs may have major roles in modulating cold-related gene expression. However, how different TFs coordinate to regulate the cold response network remains to be elucidated.

### Flavonoids, Especially Flavonols, Enhance Freezing Tolerance

Flavonoid biosynthetic genes actively respond to abiotic stress and are the determinants of freezing tolerance and CA (Schulz et al., 2016; Watkins et al., 2017). Integrating the DEMs and DEGs as well as their co-expression modules, some of the modules can be potentially linked to the biosynthesis of flavonoids, including the flavone and flavonol biosynthesis pathway and flavonoid biosynthesis pathway. In this study, 83 different flavonoids, including 26 upregulated metabolites and 7 downregulated metabolites, were detected. Here, we detected that flavonol biosynthesis genes, such as *C4H*, *CHS*, *CHI*, *F3H*, *F3’H*, and *FLS*, were all upregulated by freezing stress. The most significant enrichment was rutin in *L. spicata* leaves under freezing stress (Figures 4A–D).

Rutin is an important flavonol of medicinal value and functions as an ROS scavenger and osmotic regulator (Gęgotek et al., 2017). Analyzing mutant lines in the two *Arabidopsis* accessions that are affected in the different steps of the flavonoid biosynthetic pathway found that most flavonols and anthocyanins accumulate upon cold exposure, along with most transcripts encoding TFs and enzymes of the flavonoid biosynthetic pathway (Schulz et al., 2016), providing evidence for the functional role of flavonoids in plant CA (Schulz et al., 2016). *MYB12*, a TF gene that positively regulates rutin biosynthesis, is greatly induced by low temperature (Pandey et al., 2012). *MYB12*-overexpressing *Arabidopsis* lines show enhanced cold tolerance with higher root length and elevated levels of proline content and lower levels of malondialdehyde under cold stress conditions compared to wild-type plants (Zhou et al., 2015). In addition, flavonols also protect plants from other abiotic stresses to maintain survival. For example, the accumulation of flavonols can be promoted by MdHSFA8a, a drought-responsive HSF, contribute to ROS scavenging, and help plants survive under drought conditions (Wang et al., 2020). When *Arabidopsis* is exposed to broadband UV-B, the flavonol biosynthesis pathway responds to UV-B stress and increases accumulation, promptly allowing plants to switch from growth to UV-B stress responses (Liang T. et al., 2020). However, the molecular network of flavonols that enhance *L. spicata* freezing tolerance and whether it is suitable as a marker metabolite of freezing tolerance remain to be explored in the future.

### Abscisic Acid and Nitric Oxide May Respond to Freezing Stress

Abscisic acid plays versatile functions in regulating plant development and tolerance to various biotic and abiotic stresses. The responses of plants to abiotic stresses are mediated mainly by ABA-dependent and ABA-independent signaling pathways, which are also intertwined (Lee and Seo, 2015). In *Arabidopsis*, the MYB96–HHP module integrates cold and ABA signaling to activate the CBF-COR pathway and response to freezing stress (Lee and Seo, 2015). In rice (*Oryza sativa*), ABA receptor PYL10 (Pyrabactin Resistance 1-like 10) overexpressing transgenic lines increase cold tolerance by maintaining a higher Chl content and membrane stability index and enriching a lower amount of H_2_O_2_ (Verma et al., 2019). In chilling-sensitive rice cells and seedlings, exogenous ABA can induce some levels of freezing tolerance, probably by eliciting mechanisms different from low temperature-induced CA (Shinkawa et al., 2013). In our study, the increase in ABA biosynthesis and signaling pathway gene expression and ABA content suggests that ABA is involved in the response to the freezing stress in *L. spicata* (Figures 5A–D).

Nitric oxide signaling may also respond to the freezing stress in *L. spicata*. The oxidative pathway of NO synthesis was detected, and the key gene *NOS* was upregulated under freezing stress (Figures 6A,B). A study with a *nos1*/*noa1* mutant showed that NO negatively regulates the activities of Chl catabolic enzymes, such as PAO, resulting in the preservation of Chl degradation and the maintenance of the stability of thylakoid membranes (Liu and Guo, 2013). Notably, we detected that the expression of *NOS* was upregulated and *PAO* was downregulated in *L. spicata* leaves, which maintained green color under freezing stress (Figures 1C,E, 6B,C). NO helps plants respond and survive under abiotic stress and is related to its interaction with ROS, the modulation of gene expression and protein activity (Simontacchi et al., 2015). Moreover, NO plays a role in regulating the biosynthesis of flavonols and inhibiting the closure of stomata (Li X. et al., 2017). Given the important roles of NO in regulating plant growth and activating stress tolerance mechanisms in most plants, NO is considered as a potential tool for use in improving the yield and quality of crops (Sun et al., 2021).

### Flavonoids, Abscisic Acid, and Nitric Oxide May Play a Synergistic Effect to Comodulate Freezing Tolerance

Is there any connection among flavonoids, ABA, and NO in abiotic stress tolerance? A previous study found that ABA induces NO release in guard cells, and then NO inactivates SnRK2.6 to negatively regulate ABA signaling by S-nitrosylation of SnRK2.6 at a cysteine residue, resulting in kinase catalytic site closure and stomatal opening (Wang et al., 2015). However, NO is unlikely to be a key factor in ABA-induced rapid stomatal closure, but it allows fine tuning of stomatal aperture *via* different pathways (Van Meeteren et al., 2020). The regulation and feedback regulation between ABA and NO finely modulate stomatal closure under stress. In tea (*C. sinensis* L.) leaves, an increase in the endogenous concentration of NO induces the enrichment of flavonoids, and exogenous NO also increases flavonoids and NO levels dose-dependently (Li X. et al., 2017). Under stress, antioxidant flavonols accumulate in guard cells to scavenge ROS and maintain homeostasis. However, ABA induces stomatal closure by increasing ROS in guard cells. Studies on tomatoes show that flavonols block the ABA-dependent ROS burst and facilitate stomatal opening to modulate leaf gas exchange (Watkins et al., 2017).

**FIGURE 7:**
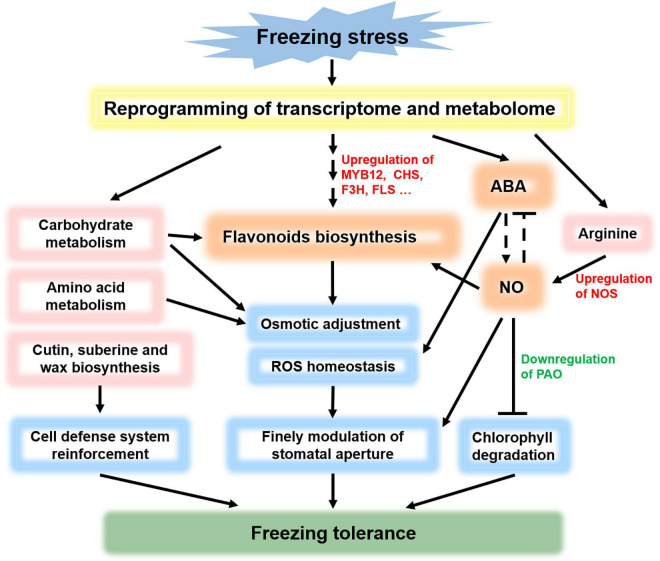
Schematic model of flavonoid, ABA, and NO comodulated freezing tolerance. Under freezing stress, the transcriptome and metabolome are reprogrammed, causing significant responses in flavonoid biosynthesis, signal transduction (including ABA and NO signals), carbohydrate metabolism, amino acid metabolism, and cutin, suberin, and wax biosynthesis. Osmotic and reactive oxygen species (ROS) are adjusted to maintain homeostasis, and the cell defense system is reinforced. Flavonoids, ABA, and NO form a regulatory network and play a synergistic effect in osmotic adjustment, ROS homeostasis, and stomatal closure. Meanwhile, *PAO* is downregulated by NO to prevent Chl degradation. In summary, the network formed by the above physiological and biochemical pathways comodulates the freezing tolerance in *L. spicata*.

In summary, we propose a model in which flavonoids, ABA, and NO comodulate the freezing tolerance in *L. spicata* (Figure 7). Under freezing stress, the transcriptome and metabolome are reprogrammed, following the main significant response pathways of flavonoid biosynthesis, ABA biosynthesis, signal transduction, carbohydrate metabolism, amino acid metabolism, and cutin, suberin, and wax biosynthesis. Osmotics and ROS are adjusted to maintain homeostasis, and the cell defense system is reinforced. Flavonoids, ABA, and NO, which have all enhanced accumulation, may form a regulatory network and play a synergistic effect in osmotic adjustment, ROS homeostasis, and stomatal closure. Meanwhile, *PAO*, the key enzyme gene of Chl degradation, is downregulated by NO, resulting in the leaves remaining green under freezing stress. Overall, the network formed by the above physiological and biochemical pathways comodulates the freezing tolerance in *L. spicata*.

## Conclusion

In this study, the potential mechanism of the freezing tolerance of *L. spicata* was explored through the integration of metabolome and transcriptome studies. A total of 581 DEMs and 10,444 DEGs, including 830 TFs, were identified in leaves. The prominent response pathways of flavonoid biosynthesis, ABA biosynthesis and signal transduction, and NO signaling were characterized. The metabolite with the most significant increase in enrichment was rutin. The genes associated with flavonol biosynthesis and ABA biosynthesis, as well as the *NOS* gene, were found to be strongly increased by freezing stress. However, the Chl degradation gene PAO was significantly decreased. Furthermore, flavonols, ABA, and NO coordinate with each other to regulate osmotic adjustment, ROS homeostasis, and stomatal closure in leaves for freezing tolerance. In summary, our results provide a network of flavonoids, ABA, and NO comodulating the freezing tolerance in *L. spicata*.

## Data Availability Statement

Data supporting the findings in this study are included in the article and Supplementary Tables. Further inquiries can be directed to the corresponding authors. The raw RNA-seq data generated in this study have been deposited in the National Center for Biotechnology Information (NCBI) Sequence Read Archive (SRA) database under accession number PRJNA761519.

## Author Contributions

L-SD conceived and designed the study. Y-YS edited the manuscript. YZ contributed to data analysis. ZP conducted the bioinformatic work and wrote the first draft. YW, W-TZ, and Y-RG performed the experiments. R-ZL, C-XY, and Z-YL revised the manuscript. All authors discussed the results and agreed to the published version of the manuscript.

## Conflict of Interest

The authors declare that the research was conducted in the absence of any commercial or financial relationships that could be construed as a potential conflict of interest.

## Publisher’s Note

All claims expressed in this article are solely those of the authors and do not necessarily represent those of their affiliated organizations, or those of the publisher, the editors and the reviewers. Any product that may be evaluated in this article, or claim that may be made by its manufacturer, is not guaranteed or endorsed by the publisher.

## Footnotes

1
http://itak.feilab.net/cgi-bin/itak/index.cgi

2
https://www.kegg.jp/kegg/pathway.html
